# Intention Understanding in Autism

**DOI:** 10.1371/journal.pone.0005596

**Published:** 2009-05-18

**Authors:** Sonia Boria, Maddalena Fabbri-Destro, Luigi Cattaneo, Laura Sparaci, Corrado Sinigaglia, Erica Santelli, Giuseppe Cossu, Giacomo Rizzolatti

**Affiliations:** 1 Dipartimento di Neuroscienze, Università di Parma, Parma, Italy; 2 Dipartimento di Scienze Biomediche e Terapie Avanzate, Università di Ferrara, Ferrara, Italy; 3 Centro Interdipartimentale Mente/Cervello (CIMeC) - University of Trento, Trento, Italy; 4 Dipartimento di Filosofia, Università degli Studi di Milano, Milano, Italy; 5 Neuropsichiatria Infantile, Azienda Unità Sanitaria Locale di Reggio Emilia, Reggio Emilia, Italy; King's College London, United Kingdom

## Abstract

When we observe a motor act (e.g. grasping a cup) done by another individual, we extract, according to how the motor act is performed and its context, two types of information: the goal (grasping) and the intention underlying it (e.g. grasping for drinking). Here we examined whether children with autistic spectrum disorder (ASD) are able to understand these two aspects of motor acts. Two experiments were carried out. In the first, one group of high-functioning children with ASD and one of typically developing (TD) children were presented with pictures showing hand-object interactions and asked *what* the individual was doing and *why*. In half of the “why” trials the observed grip was congruent with the function of the object (“why-use” trials), in the other half it corresponded to the grip typically used to move that object (“why-place” trials). The results showed that children with ASD have no difficulties in reporting the goals of individual motor acts. In contrast they made several errors in the *why* task with all errors occurring in the “why-place” trials. In the second experiment the same two groups of children saw pictures showing a hand-grip congruent with the object use, but within a context suggesting either the use of the object or its placement into a container. Here children with ASD performed as TD children, correctly indicating the agent's intention. In conclusion, our data show that understanding others' intentions can occur in two ways: by relying on *motor* information derived from the hand-object interaction, and by using *functional* information derived from the object's standard use. Children with ASD have no deficit in the second type of understanding, while they have difficulties in understanding others' intentions when they have to rely exclusively on motor cues.

## Introduction

Autistic spectrum disorder (ASD) is a heterogeneous syndrome characterized by impairment in social skills, verbal and nonverbal communication, and restricted and repetitive behaviors [1]. Deficits in the domains of affective links and emotional behavior are other aspects of ASD [2]–[4].

Autism affects a variety of nervous structures ranging from the brainstem to the cerebellum and the cerebral cortex [5]–[11]. As far as the cerebral cortex is concerned, evidence has been recently provided for a marked disorder of its connectivity involving primarily, although not exclusively, intrahemispheric connections [see 12]–[16]. Beside white matter, alterations of gray matter and its intrinsic connectivity have also been reported [17]–[20]. Among studies reporting gray matter alterations, of particular interest is the study showing a correlation between the thinning of fronto-parietal areas and the severity of autistic impairment [20]. Alterations of cortical connectivity and, in particular, of association areas have been proposed to represent one of the major causes, or possibly the major cause, of the cognitive deficits characterizing ASD [16], [21].

These cortical abnormalities appear to affect the functioning of mirror mechanism [20], a neural mechanism that plays an important role in social cognition [22]–[26]. Evidence for mirror mechanism impairment in ASD comes from EEG [27]–[32], MEG [33], TMS [34] and fMRI studies [35]. Among them, particularly influential in establishing a link between mirror mechanism impairment and autistic disorders has been an fMRI study by Dapretto et al [35]. These authors scanned high functioning children with ASD and matched controls during imitation and observation of emotional expressions. The results showed a significantly weaker activation in the inferior frontal gyrus in children with ASD with respect to TD children. Most interestingly, the activation was inversely related to symptom severity.

While instrumental data indicate a deficit in the mirror mechanism in autism, behavioral findings appear to challenge this link [36]–[39]. In particular, a recent study that specifically tested the “mirror mechanism hypothesis” of ASD found that children with ASD recognize the goal of others' motor acts, a function that, according to the standard interpretation of the mirror mechanism, has to be impaired in the case of mirror mechanism malfunctioning [36].

To get an insight into the possible reasons for this discrepancy between behavioral and instrumental data, it is important to make clear that the term “action understanding” conceals two different meanings. An example will clarify this point: John observes Mary who is grasping a cup of coffee. John immediately understands two things: a) the *what* of Mary's motor act (she is grasping the cup) and b) the *why* of Mary's motor act (e.g. she is grasping the cup to drink coffee). These two aspects of action understanding, although frequently confused, are actually radically different one from the other. The first provides an *immediate* perceptual datum derived by motor act observation; the second is an *anticipation* of a future behavior based on an “intention-reading” mechanism. There is evidence that the mirror mechanism is involved in both these aspects of action understanding [40], but the way in which it is involved is different in the case of the *what* and the *why* of a motor act.

The *what* of a motor act (e.g. grasping) derives from the activation of mirror neurons which determines in the cortical motor system of the observer a motor representation matching the observed motor act. This motor representation allows the observer to know what the other is doing.

This mechanism, however, does not appear to be sufficient to allow one to understand the *why* of an observed motor act. The why requires a more complex mechanism, which, although centered on mirror neurons, also involves other motor neurons. It has been recently shown that in the inferior parietal lobule (IPL) there is a set of neurons (‘action-constrained motor neurons’) that fire only when a motor act (e.g. grasping) is part of a given action (e.g. grasping for eating) [41]. These neurons are organized into chains, where each neuron codes a certain motor act (e.g. reaching, grasping, etc.). When an individual intends to perform a given action (e.g. to reach a piece of food to eat it) an entire chain is activated, leading to the fulfillment of his/her intention. Most interestingly, many action-constrained motor neurons also fire during action observation. This activation, induced by the observed motor act, triggers the same action chain that the observers endogenously activate to achieve their intention. This mechanism enables the observer to understand directly the motor intention of others without inferential processing.

The distinction between single neuron- and action chain-based mirror mechanisms might provide a solution to the present contradiction between the neurophysiological data showing a deficit of the mirror mechanism in autism and the behavioral data indicating that the understanding of the goal of a motor act is intact in ASD. A possibility is that the basic single neuron mirror mechanism is essentially intact in ASD, but the chained organization is impaired.

Cattaneo et al. [42] provided evidence that chaining is impaired in ASD. They studied a group of TD children and a group of children with ASD while they observed an experimenter grasping an object with two different purposes, to eat or to place it into a container. The EMG activity of the mylohyoid muscle (MH), a muscle involved in mouth opening, was recorded. The results showed that in TD children, the observation of grasping leading to eating determined an activation of the MH muscle, while such activation was not present in children with ASD. In a second experiment both ASD and TD children were asked to perform the same actions. In TD children activation of the MH muscle started as soon as they began the reaching movement, much before the object was grasped. In contrast, no MH muscle activation was observed during reaching and grasping in children with ASD. MH muscle activation appeared only late, when children started bringing food to the mouth.

These data indicate, on the one side, that children with ASD are impaired in assembling their individual motor acts (reaching, grasping, placing) into a unitary action characterized by a specific intention (e.g. grasping-for-eating), on the other that their mirror chains are weakened, as shown by the lack of MH muscle activation (recorded in TD children) during action observation.

Given these findings, the question arises of whether the behavioral data that suggest an intact mirror mechanism in ASD children derives from the fact that, in those studies, only the *what* aspect of action understanding was tested or whether indeed children with ASD have no deficits in both aspects (the *what* and the *why*) of action understanding. To answer this question we carried out two behavioral studies in which TD and ASD children were asked to watch hand-object interactions and to identify the observed motor acts as well the motor intention underlying them. The results showed that children with ASD are able to understand the *what* of a motor act, but are impaired in understanding the *why* of it when they have to rely exclusively on the agent's motor behavior.

## Methods

This study consists of two experiments. They were carried out on a group of children with ASD (15 males and 1 female, mean age: 9.74±2.22) and a group of typically developing (TD) children (21 males and 4 females, mean age: 8.34±0.57). The experiments were approved by the local ethical committee and were conducted according to the Helsinki declaration. The parents of the participants gave informed written consent.

Children with ASD were recruited at the Center for Pediatric Neuropsychiatry in Empoli (ASL 11) and at the Center for Autism in Reggio Emilia. The diagnosis was made by a licensed clinical psychologist or a medical doctor not associated with this research. Module 3 of the Autism Diagnostic Observation Schedule (ADOS) was used to confirm the diagnosis of autistic disorder or autism spectrum disorder [43]. Scores from 7 to 10 (Module 3) indicate autistic spectrum disorder and scores from 10 and above indicate autism. The mean ADOS total score was of 14.5 (SD 3.77). Based on the results of this scale and clinical judgment, 14 of the 16 children met criteria for autistic disorder, and the remaining 2 met criteria for autism spectrum disorder. All the patients had an intelligence quotient (IQ)≥70, calculated with the Wechsler Intelligence Scale for Children-Revised (WISC-R) [44] with mean IQ 88.18 (SD 12.28). Table 1 shows age, IQ, and ADOS values for all children of the ADS group.

**Table 1:** Demographics for children participating in the study.

	ASD Group (N = 16)	TD Group (N = 25)
	(Mean/SD)	(Mean/SD)
**Chronological Age**	9.74 (+/−2.22)	8.34 (+/−0.57)
**IQ**	88.18 (+/−12.28)	NA
**Non Verbal Cognitive Level**	78.00 (+/−20.16)	88.12 (+/−15.56)
**Verbal Age**	11.11 (+/−4.40)	11.95 (+/−3.41)
**ADOS**	14.5 (+/−3.77)	NA

The control group was matched to the ASD group for verbal age, evaluated by Peabody Picture Vocabulary test (PPVT-R) [45] and for non-verbal cognitive level, tested by Raven's Progressive Matrices [26]. The mean score of Raven's Progressive Matrices did not differ significantly (p = 0.49) between the two groups. The mean values were 78.00 (SD 20.16) for the ASD group and 88.12 (SD 15.56) for the TD group. Mean verbal ages as assessed by the Peabody Picture Vocabulary test (PPVT) were also not significantly different (p = 0.55) in the two groups. Mean values were 11.11 years (SD 4.40) for the ASD group and 11.95 years (SD 3.41) for the TD.

### Experiments 1

**Figure 1:**
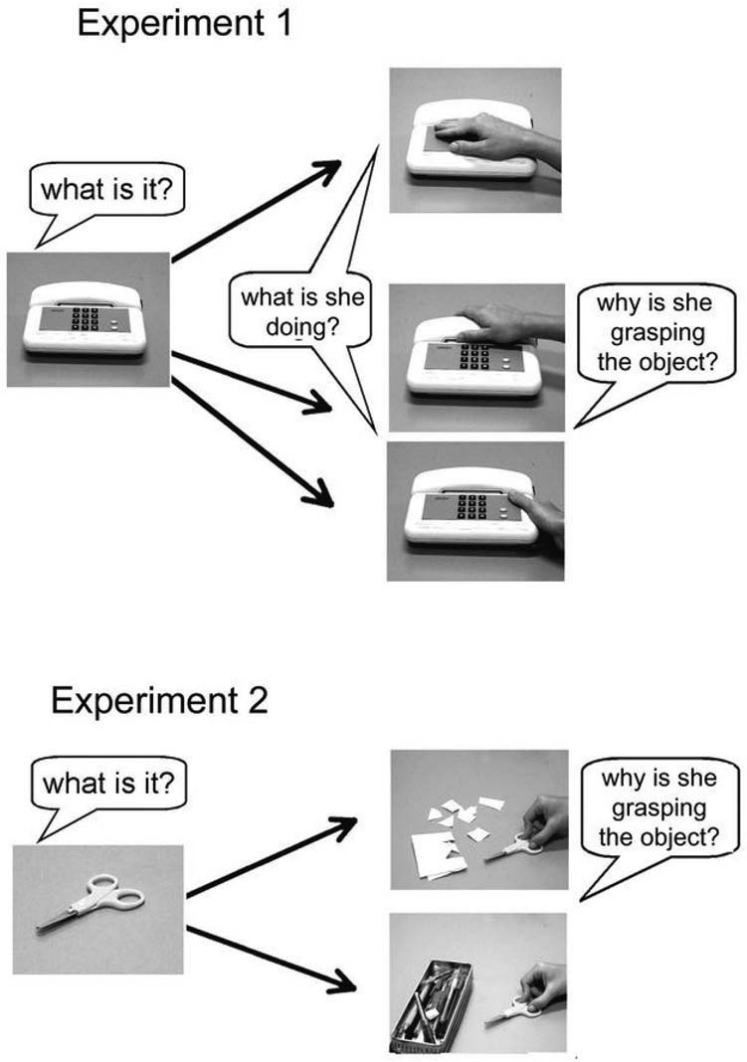
Experimental design of the two experiments. Examples of stimuli employed and the relative questions are shown.

Children saw two pictures presented one after the other on a computer screen (Figure 1, top). The first picture showed an object on a neutral background. Participants were asked to name it and their response was recorded. A second picture was then presented, showing the same object plus a hand making contact with it. The transition between the two pictures was done manually. The second picture showed one of the following three types of hand-object interactions: a) a hand touching an object (“touch” pictures); b) a hand grasping an object with a grip commonly employed for moving and placing it to another location (“place” pictures); c) a hand grasping an object with the grip typically employed for using that object (“use” pictures). The total set of stimuli for each child comprised 17 objects ×3 hand-object interaction pictures for a total of 51 stimulus pairs. They were presented in a pseudo-random order.

A brief training session of 7 stimulus pairs preceded the experiment. During training, when viewing the second picture the child was asked: “What is she doing? *Touching or grasping*?” When the picture showed a grip and the child correctly answered “grasping”, the experimenter further asked: “*Why* is she grasping the object? *To place it or to use it*?” During the experimental session the questions asked to the children did not include the forced choice provided in the training, instead they were asked: “*What is she doing*?” (“what” task) and “*Why is she grasping the object*?” (“why” task). Therefore the “what” task occurred over the 51 trials, while the “why” task over 34 trials. The children were allowed to take as much time as they wanted to answer. All trials were videotaped.

Responses were categorized as correct or incorrect. Responses to the first question were considered correct if the answer was “touching” or “grasping” in response to the appropriate picture. Answers to the second question were considered correct if children reported the intention typically related to the use of the object (e.g. “to brush” for a brush, “to phone” for a telephone) in response to “use” pictures, or answered “placing” in response to “place” pictures. Separate error rates were calculated for “why-use” (17 trials) and “why-place” (17 trials) responses.

### Experiment 2

As in Experiment 1 children saw two pictures presented one after the other on a computer screen. The first picture showed an object on a neutral background. Participants were asked to name it and their response was recorded. A second picture showed a hand grasping an object. The grip was always a use grip. Near the hand, there was another object (or group of objects) that suggested either: (a) an intention of placing the object (“why-place” task; e.g. a pair of scissors grasped near a container) or (b) the intention of using it (“why-use” task; e.g. a pair of scissors grasped near a sheet of paper) (Figure 1, bottom). The total set of stimuli for each child comprised 17 objects ×2 context pictures for a total of 34 presentations. The stimulus pairs were presented in a pseudo-random order.

A brief training session made up of 7 stimulus pairs preceded the experiment. The experimental procedure was the same as in Experiment 1. Responses were rated as correct if the answer to the question matched the agent's intentions as suggested by the context. Otherwise they were labeled as incorrect. Separate error rates were calculated for each subject for “why-use” trials (17 trials) and for “why-place” trials (17 trials).

### Statistical Analysis

Comparisons between the demographical data and clinical test scores of the two groups were performed with t-tests. The main analysis was conducted with ANOVAs on error rates as dependent variable. In Experiment 1 a mixed ANOVA with two factors was carried out: between-subjects factor, Group, (2 levels: TD and ASD group), within-subjects factor, Task type, (3 levels: “what” task, “why-use” task and “why-place” task).

In Experiment 2 a mixed ANOVA with two factors was performed employing a between-subjects factor, Group, (2 levels: TD and ASD group) and a within-subjects factor, Task type (2 levels: “why-use” and “why-place”).

Post-hoc analysis in both experiments was carried out using multiple t-tests with Bonferroni correction. Confidence intervals for differences between means have also been assessed.

## Results

### Experiment 1

**Figure 2:**
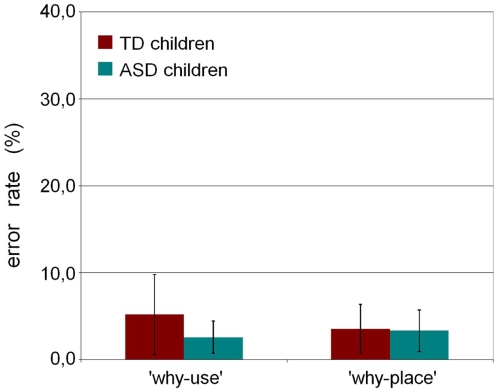
Results of Experiment 1. Error rates are plotted as percentage for each task. *** indicates significant difference (p = 0.0001). Error bars represent 95% CI.

The percentage error for TD and ASD children in “what”, “why-use” and “why-place” trials are shown in Fig. 2. The number of errors in the first two types of trials (“what” and “why-use”) is similar across both groups. In contrast, the number of errors in “why place” trials is markedly higher in ASD group. Table 2 shows the individual error rates, expressed as percentages, for every participant and their IQ.

**Table 2:** Individual error rates expressed as percentages for all participants in the Experiment 1.

n.	Group	IQ	What	Why-place	Why-use
1	ASD	102	15.7	32.4	2.9
2	ASD	75	2.0	41.2	0.0
3	ASD	94	15.7	26.5	0.0
4	ASD	102	0.0	41.2	0.0
5	ASD	87	11.8	5.9	0.0
6	ASD	77	5.9	8.8	5.9
7	ASD	91	7.8	23.5	0.0
8	ASD	78	7.8	23.5	5.9
9	ASD	75	0.0	11.8	5.9
10	ASD	87	2.0	14.7	2.9
11	ASD	90	15.7	29.4	0.0
12	ASD	70	5.9	29.4	2.9
13	ASD	72	2.0	38.2	2.9
14	ASD	89	0.0	47.1	0.0
15	ASD	91	11.8	14.7	14.7
16	ASD	110	0.0	11.8	11.8
1	TD	-	0.0	8.8	0.0
2	TD	-	11.8	5.9	0.0
3	TD	-	13.7	2.9	2.9
4	TD	-	0.0	5.9	0.0
5	TD	-	3.9	5.9	0.0
6	TD	-	5.9	14.7	0.0
7	TD	-	2.0	8.8	2.9
8	TD	-	2.0	23.5	8.8
9	TD	-	2.0	8.8	0.0
10	TD	-	0.0	11.8	2.9
11	TD	-	9.8	8.8	0.0
12	TD	-	2.0	0.0	0.0
13	TD	-	3.9	5.9	0.0
14	TD	-	0.0	11.8	11.8
15	TD	-	0.0	8.8	2.9
16	TD	-	2.0	8.8	17.6
17	TD	-	2.0	14.7	5.9
18	TD	-	3.9	20.6	14.7
19	TD	-	11.8	8.8	11.8
20	TD	-	2.0	2.9	14.7
21	TD	-	5.9	8.8	5.9
22	TD	-	13.7	14.7	5.9
23	TD	-	2.0	14.7	11.8
24	TD	-	3.9	2.9	0.0
25	TD	-	2.0	11.8	2.9

The ANOVA showed a significant main effect of Group (F(1, 39) = 23.1, p<0.0001), with ASD children making more errors than TD children, and Task type (F(2, 78) = 41.7, p<0.0001) with more errors in the “why-place” trials. A significant interaction was also found between the Group and the Task type factors (F(2,78) = 15.4, p<0.0001). The relevant post-hoc comparisons and the confidence intervals for the differences between means (Table 3) showed a marked increase in error rates in ASD children compared to TD children in the “why-place” task, in spite of no difference between the two groups in the error rates for the “what” and “why-use” tasks.

**Table 3:** Post-hoc comparisons in Experiment 1 between the error rates of the two groups in each of the 3 tasks.

	Mean (SD)	Mean (SD)	*t-value*	*DF*	*p-value*	*−95%*	*+95%*
	*ASD (n = 16)*	*TD (n = 25)*					
**What task**	6.50 (6.0)	4.23 (4.4)	1.386758	39	0.173392	−2.6576	7.177203
**Why-use task**	3.49 (4.5)	4.94 (5.7)	−0.863138	39	0.393339	−3.10933	6.006384
**Why-place task**	25.0 (12.8)	9.65 (5.5)	5.300345	39	0.000005	6.342845	24.36304

Correction for the number of comparisons gives a significance level of 0.016. In the last 2 columns confidence intervals for mean differences are shown.

### Experiment 2

**Figure 3:**
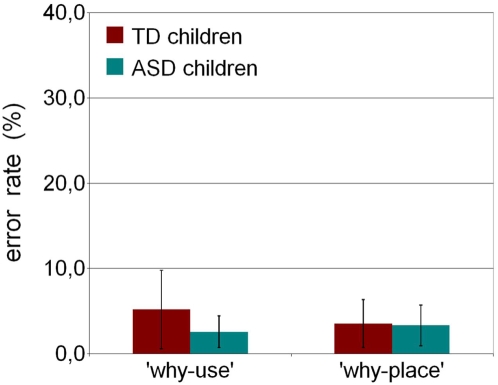
Results of Experiment 2. Error rates are plotted as percentage for each task.

The percentage of errors made by TD and ASD children in the two types of trials of this experiment are shown in Fig 3. The number of errors in both types of trials (“why-use” and “why-place”) was similar in the two groups. Table 4 shows the individual error rates, expressed as percentages, for every participant.

**Table 4:** Individual error rates expressed as percentages for all participants in the Experiment 2.

n.	Group	IQ	Why-place	Why-use
1	ASD	102	11.8	11.8
2	ASD	75	0.0	0.0
3	ASD	94	0.0	5.9
4	ASD	102	0.0	0.0
5	ASD	87	11.8	0.0
6	ASD	77	0.0	0.0
7	ASD	91	0.0	0.0
8	ASD	78	0.0	5.9
9	ASD	75	0.0	0.0
10	ASD	87	5.9	5.9
11	ASD	90	11.8	0.0
12	ASD	70	5.9	5.9
13	ASD	72	5.9	0.0
14	ASD	89	0.0	5.9
15	ASD	91	0.0	0.0
16	ASD	110	0.0	0.0
1	TD	-	0.0	29.4
2	TD	-	5.9	5.9
3	TD	-	11.8	11.8
4	TD	-	5.9	5.9
5	TD	-	0.0	0.0
6	TD	-	0.0	0.0
7	TD	-	0.0	0.0
8	TD	-	0.0	0.0
9	TD	-	11.8	11.8
10	TD	-	17.6	29.4
11	TD	-	0.0	0.0
12	TD	-	0.0	0.0
13	TD	-	5.9	23.5
14	TD	-	0.0	0.0
15	TD	-	17.6	0.0
16	TD	-	0.0	0.0
17	TD	-	5.9	5.9
18	TD	-	0.0	0.0
19	TD	-	0.0	0.0
20	TD	-	0.0	0.0
21	TD	-	0.0	5.9
22	TD	-	0.0	0.0
23	TD	-	0.0	0.0
24	TD	-	0.0	0.0
25	TD	-	5.3	5.3

The ANOVA did not show any significant effect of the Group (F (1, 39) = 0.74268, p = 0.39407) or Task (F (1, 39) = 0.25298, p = 0.61781) factors, nor did it show any interaction between the two factors (F (1, 39) = 1.3175, p = 0.25804). Post-hoc comparisons and the confidence intervals for the differences between means confirmed the absence of differences in the performance of the two groups in both tasks (Table 5).

**Table 5:** Post-hoc comparisons in Experiment 2 between the error rates of the two groups in each of the 3 tasks.

	Mean (SD)	Mean (SD)	*t-value*	*DF*	*p-value*	*−95%*	*+ 95%*
	*ASD (n = 16)*	*TD (n = 25)*					
**Why-use task**	2.57 (3.7)	5.39(9.1)	−1.16828	39	0.249784	−2.77758	8.404516
**Why-place task**	3.31 (4.7)	3.50 (5.6)	−0.11503	39	0.909008	−4.51212	4.903765

Correction for the number of comparisons gives a significance level of 0.025. In the last 2 columns confidence intervals for mean differences are shown.

## Discussion

Before proceeding to discuss the data of the present study, let us come back to the example given in the introduction: Mary and her cup of coffee. The deceptively simple action of drinking coffee is not unitary, but comprises a series of discrete steps: reaching for the cup, grasping it, holding it, and bringing the cup to the mouth. These action elements are referred to as *motor acts* [47]. Each motor act has its own goal, that of organizing the movements in such a way that the effectors may interact with the objects in an efficient way. Mary's intention selects motor acts and unifies them into a *motor action*. Her intention is fulfilled when she achieves the final goal of the action and obtains reinforcement.

Now, when witnessing the motor act performed by Mary (e.g. grasping a cup), John recognizes *what* she is doing, that is the goal of that motor act. In addition, according to how this motor act is performed, coupled with the context in which it is performed, he also understands *why* she is doing it, i.e. Mary's motor intention.

As far as the understanding of *what* is concerned, Experiment 1 showed that children with ASD have no difficulties in reporting the goal of observed motor acts. They were able to understand the hand-object interactions without any significant difference with respect to TD children. A different and more complex pattern was found for the understanding of *why*. Unlike TD children, children with ASD exhibited a significant deficit in understanding the intention underlying the observed motor act. This deficit was present, however, only in the “why-place” task, but not in the “why-use” task.

How can this discrepancy be accounted for? An explanation may be found by considering the two different types of information on the agent's intention, that the observation of a hand grasping an object provides to an observer: a) *motor* information, based on the observed hand-object interaction and b) *functional* information, based on the object's typical use. In the “why-use” task the motor information was congruent with the functional one; therefore, both the handgrip and the object function suggested the same intention. By contrast, in the “why-place” task such congruence was lacking, and intention understanding must rely exclusively on the hand-object motor interaction. The increased error rates for children with ASD, with respect to TD children, in the “why-place” trials indicate, therefore, that, unlike TD children, children with ASD did not fully succeed in processing the motor information coming from the agent's hand shape, and based their judgment concerning the agent's intention mainly on the object's functional information. Thus, the sight of a cup triggered the response “for drinking”, while the sight of a pair scissors the response “for cutting”, even when the observed handgrip rendered these actions very implausible.

The results of Experiment 2 corroborated this interpretation. In this experiment, the handgrip was congruen*t with object use* in both the “why-use” and “why-place” trials, but other objects with specific functions provided additional information on the agent's intention (e.g. scissors near a piece of paper = cutting; scissors near a box = putting the scissors into the box). The rational of the experiment was the following: if children with ASD fail in intention understanding when they have to rely on motor information, the substitution of motor cues (i.e. different kinds of hand-grip) with functional cues (i.e. additional objects having different functions located close to the object the hand interacts with) should allow children with ASD to markedly improve their performance in the “why-place” task, bringing it to the level reported in the “why-use” task. This was exactly what was found. In Experiment 2 children with ASD performed both the “why” tasks with very low error rates, recognizing the agent's intention in the same way as TD children.

Experiment 2 also shows that the errors of children with ASD in the “why-place” trials in Experiment 1 were not due to stereotyped responses triggered by the objects, or to some intellectual deficit, but to their incapacity to use motor information to understand the intention of others. When they had additional information from objects surrounding the object acted upon in “why-place” trials, they were able to perform the task and read correctly the agent's intention to place the object rather than using it.

Taken together, these findings allow one to offer an explanation for the apparent contradiction between, on the one side, electrophysiological and brain imaging data suggesting that a deficit in the mirror mechanism could be the basis for autistic impairment in action understanding [27]–[35] and, on the other, behavioral studies indicating that children with ASD do not present deficits in understanding observed motor acts [36]–[39]. The “what” task in our Experiment 1 showed that children with ASD are able to recognize individual motor acts with the same error rates as TD children. This does not imply, however, that the “mirror mechanism hypothesis” of ASD is wrong. As mentioned in Introduction, neurophysiological data indicate a clear distinction between single neuron-based mirror mechanism and chain-based mirror mechanism in action understanding. The first mechanism plays a fundamental role in understanding what individuals are doing, the other why they are doing it, i.e. their motor intention. Although, we cannot exclude the possibility that the single-neuron mirror mechanism is hypofunctioning in children with ASD (bearing in mind the extreme simplicity of our tasks) yet the main deficit appears to depends on a deficit in the chain-based mirror mechanism.

Consistent with this interpretation are the data from the study of Cattaneo et al. [42]. This study showed that, unlike TD children, children with ASD show a deficit in translating their intentions into motor actions as well as in activating, during the observation of others' actions, their own corresponding motor chains. These findings clearly indicate that the chained organization of motor acts is impaired in children in ASD. They also show that children with ASD do not execute motor tasks using intention-based anticipatory behavior, as TD children do, but their action organization relies step-by-step on functional characteristics of the objects they act upon. This behavior parallels the tendency shown by children with ASD in the present experiment to interpret the behavior of others on the basis of the functional information given by objects, rather than on the intentional information present in their motor acts.

Interpretation of the “mirror hypothesis” of ASD in terms of a deficit of the chain-based mirror mechanism [42], rather than in terms of hypo-function of mirror neurons [see 22], [25], [27]–[35] is in accord with growing evidence of alterations in intra-hemispheric connectivity in ASD [see Introduction] and the proposal that these alterations represent a major cause of the cognitive deficits in ASD [16], [21]. Although intra-hemispheric alterations may cause a deficit in the development of individual mirror neurons due, for example, to weakened connections between the superior temporal sulcus areas (where neurons with complex visual properties are located, see [48]), and the inferior parietal lobule (a core center of the mirror system for non-emotional actions, see [24], these alterations ought to produce more destructive effects on the chained organization of the mirror system, which implies a complex network, than on individual mirror neurons. Furthermore, the hypothesis of a deficit of mirror neurons *per se* has difficulties in accounting for impairment in the organization of actions during their execution in ASD children [42], while by contrast this impairment fits well with the notion of diffuse connectivity alterations.

The demonstration of a deficit in intention understanding based on motor information does not imply of course that children with ASD are unable to grasp the intentions of others at all. The capacity to understand others' intentions can be also mediated by other mechanisms. It could derive from the functional use of the objects the agent interacts with (see Experiment 1), from the objects surrounding those objects (Experiment 2), and possibly, in some circumstances, as in TD individuals, from inferential mechanisms [49]–[51]. Guessing others' intentions, however, on the basis of the functional use of objects provides only a rigid and often unreliable way of understanding others. It may be that inferential processing based on additional contextual or social information present in the environment could help children with ASD to overcome the pitfalls of an object-based intention guessing mechanism. However, even with this additional inferential processing the comprehension of others could hardly reach the reliability and, especially, the effortlessness typical of action understanding based on one's own motor competence. One may also wonder whether, without motor understanding of others, individuals could have those experiential aspects of what others are doing that are fundamental for establishing a satisfactory social life. This hypothesis, however, although not unlikely, requires further empirical exploration.
