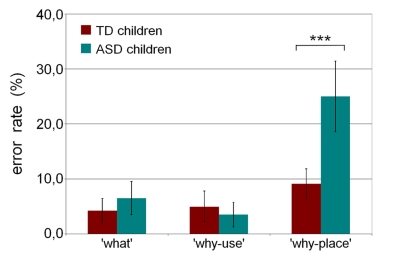# Correction: Intention Understanding in Autism

**DOI:** 10.1371/annotation/3f865d29-8d14-4f15-86dc-061631ff6d78

**Published:** 2009-06-13

**Authors:** Sonia Boria, Maddalena Fabbri-Destro, Luigi Cattaneo, Laura Sparaci, Corrado Sinigaglia, Erica Santelli, Giuseppe Cossu, Giacomo Rizzolatti

Figure 2 is incorrect. The correct figure can be viewed at: 

**Figure:**